# Measuring Physical Activity in Free-Living Conditions—Comparison of Three Accelerometry-Based Methods

**DOI:** 10.3389/fphys.2016.00681

**Published:** 2017-01-10

**Authors:** Anna-Maiju Leinonen, Riikka Ahola, Janne Kulmala, Harto Hakonen, Henri Vähä-Ypyä, Karl-Heinz Herzig, Juha Auvinen, Sirkka Keinänen-Kiukaanniemi, Harri Sievänen, Tuija H. Tammelin, Raija Korpelainen, Timo Jämsä

**Affiliations:** ^1^Research Unit of Medical Imaging, Physics and Technology, University of OuluOulu, Finland; ^2^Infotech Oulu, University of OuluOulu, Finland; ^3^Department of Sports and Exercise Medicine, Oulu Deaconess InstituteOulu, Finland; ^4^Medical Research Center, Oulu University Hospital and University of OuluOulu, Finland; ^5^LIKES - Research Center for Sport and Health SciencesJyväskylä, Finland; ^6^UKK Institute for Health Promotion ResearchTampere, Finland; ^7^Research Unit of Biomedicine, University of OuluOulu, Finland; ^8^Department of Gastroenterology and Metabolism, Poznan University of Medical SciencesPoznan, Poland; ^9^Biocenter Oulu, University of OuluOulu, Finland; ^10^Center for Life Course Health Research, University of OuluOulu, Finland; ^11^Diagnostic Radiology, Oulu University HospitalOulu, Finland

**Keywords:** accelerometer, agreement, middle-aged, objective measurement, sedentary time

## Abstract

We examined the agreement in time spent on different physical activity (PA) levels using (1) mean amplitude deviation (MAD) of raw acceleration from the hip, (2) wrist-worn Polar Active, and (3) hip-worn Actigraph counts using Freedson's cut-points among adults under free-living conditions. PA was measured in 41 volunteers (mean age 47.6 years) for 14 days. Two MET-based threshold sets were used for MAD and Polar Active for sedentary time (ST) and time spent in light (LPA), moderate (MPA), and vigorous (VPA) PA. Actigraph counts were divided into PA classes, ≤100 counts/min for ST and Freedson's cut-points for LPA, MPA, and VPA. Analysis criteria were simultaneous use of devices for at least 4 days of >500 min/d. The between-method differences were analyzed using a repeated measures analysis of variance test. Bland-Altman plots and ROC graphs were also employed. Valid data were available from 27 participants. Polar Active produced the highest amount of VPA with both thresholds (≥5 and ≥6 MET; mean difference 17.9–30.9 min/d, *P* < 0.001). With the threshold 3–6 MET for MPA, Polar Active indicated 19.2 min/d more than MAD (95% CI 5.8–32.6) and 51.0 min/d more than Actigraph (95% CI 36.7–65.2). The results did not differ with 3.5–5 MET for MPA [*F*_(1.44, 37.43)_ = 1.92, *P* = 0.170]. MAD and Actigraph were closest to each other for ST with the threshold < 1.5 MET (mean difference 22.2 min/d, 95% CI 7.1–37.3). With the threshold <2 MET, Polar Active and Actigraph provided similar results (mean difference 7.0 min/d, 95% CI −17.8–31.7). Moderate to high agreement (area under the ROC curve 0.806–0.963) was found between the methods for the fulfillment of the recommendation for daily moderate-to-vigorous PA of 60 min. In free-living conditions the agreement between MAD, Polar Active, and Actigraph for measuring time spent on different activity levels in adults was dependent on the activity thresholds used and PA intensity. ROC analyses showed moderate to high agreement for the fulfillment of the recommendation for daily MVPA. Without additional statistical adjustment, these methods cannot be used interchangeably when measuring daily PA, but any of the methods can be used to identify persons with insufficient daily amount of MVPA.

## Introduction

Regular physical activity (PA) reduces the risk of many diseases, such as cardiovascular diseases, metabolic syndromes, type 2 diabetes, certain cancers, and depression (McGuire and Ross, 2011; Wen et al., 2011; Ekelund et al., 2012; Lee et al., 2012; Sieverdes et al., 2012). On the contrary, sedentary behavior has recently been suggested to pose even greater health risk than obesity (Ekelund et al., 2015). Despite the growing evidence for the positive health effects of PA, our lifestyle is becoming more inactive worldwide (Hallal et al., 2012). To improve specification of the amount of health enhancing PA, objective methods to measure PA, including sedentary time, are needed.

Nowadays, several types of accelerometers and analysis methods to measure the intensity, frequency, and duration of PA and sedentary time are available. The challenge is that the output of different methods varies and there still exists lack of consensus which of the methods provides the accurate results (Welk et al., 2012). Based on studies in laboratory, in short-term free-living conditions or under structured protocols, the output seems to be related to the used algorithms and thresholds of different PA levels (Strath et al., 2003; Reilly et al., 2008; Trost et al., 2011; Crouter et al., 2013; Thiese et al., 2014; Watson et al., 2014). Recently, the use of the accelerometers that provide raw acceleration signal instead of some specific unit (e.g., counts) has increased. However, transparent algorithms to analyse raw acceleration signal are still missing (Troiano et al., 2014).

Mean amplitude deviation (MAD) is a recently proposed universal method to analyse raw triaxial acceleration signals (Aittasalo et al., 2015; Vähä-Ypyä et al., 2015a,b). A strong correlation (*R*^2^ = 0.94) has been found between incident MAD measured with a waist-worn device (Hookie AM20, Traxmeet, Finland) and oxygen consumption in walking and running (Vähä-Ypyä et al., 2015a). For the epoch of interest, the MAD is calculated from the resultant of the three orthogonal accelerations and it describes the mean variation of the dynamic acceleration component around the static component. On the other hand, Polar Active represents wrist-worn accelerometer with high user-friendliness (Brugniaux et al., 2010; Schaefer et al., 2014; Troiano et al., 2014; Jauho et al., 2015). In earlier studies Polar Active has been found to relate to indirect calorimetry and double labeled water with regard to energy expenditure (EE) calculation (Brugniaux et al., 2010; Kinnunen et al., 2012). Actigraph is currently the most widely used accelerometer for research purposes (Robusto and Trost, 2012; Santos-Lozano et al., 2012; Troiano et al., 2014). In previous studies, several Actigraph prediction equations for estimating EE have been developed (Crouter et al., 2006).

Although, there are some studies on convergent validity between accelerometry-based methods used in research (Welk et al., 2000; Paul et al., 2007; Esliger et al., 2011; John et al., 2013; Hildebrand et al., 2014; Lee et al., 2015; Rowlands et al., 2015), further equivalency studies are needed to better cover the wide range of available measurement methods. The above-mentioned three different accelerometer-based PA measurement methods have not been compared so far. Thus, the present study aims to produce this missing knowledge by comparing these methods, including one method developed to analyze raw acceleration data. Accordingly, the aim of this study was to examine the convergent validity in measured time spent on different PA levels calculated with MAD using raw acceleration data, Polar Active, and the Freedson equation for Actigraph counts among adults in free-living conditions.

## Materials and methods

### Participants

The study is a part of the pilot study of 46-year data collection of The Northern Finland Birth Cohort 1966 (NFBC1966) (Rantakallio, 1988). For the pilot study, 150 invitations were sent to adults selected randomly from the national population register, born in 1964–65 and living in Oulu area or in neighboring municipalities. A total of 45 respondents were recruited, their mean age being 47.6 (SD 0.6) years. In the beginning, the study participants were invited to a baseline visit, where their body mass and height were measured and they received the accelerometers with oral and written instructions. In addition, the participants received a prepaid-postage padded envelope for returning the devices by mail.

Of the 45 participants recruited, 41 (91%) attended the baseline visit and agreed to use the three accelerometers for 2 weeks. Simultaneous activity data comprising at least four valid days from all three devices were available from 29 (71%) participants. Three participants wore the monitors for 1 day only, in four cases the battery of one of the monitors had discharged, and in five cases one of the monitors had malfunctioned and the data were not recorded. During the analyses, two cases with invalid data were observed and were excluded from further analyses. In the end, 27 (66%) participants were included in the analyses. The characteristics of the 27 participants are shown in Table 1.

**Table 1:** **Characteristics of the study participants included in the analyses**.

	**Females (*n* = 20)**	**Males (*n* = 7)**
Age [years]	47.6 (0.6) [47−49]	47.7 (0.7) [47−49]
Height [cm]	164.6 (6.3) [150.8−176.0]	177.7 (6.7) [165.1−189.0]
Body mass [kg]	72.6 (13.6) [51.6−102.3]	80.9 (8.3) [65.1−89.5]
BMI [kg/m^2^]	26.7 (4.6) [18.9−38.6]	25.6 (2.4) [20.9−28.5]

*Values are mean (SD) [range]*.

All participants were asked for a written consent to take part in the study. Prior to obtaining consent, the participants were given both oral and written information about the study and their right to refuse to take part or to withdraw from the study. The study followed the legislation decrees and ethical principles concerning medical research on humans in Finland and the Declaration of Helsinki. The permission for the NFBC1966 study was received from the Ethics Committee of Northern Ostrobothnia Hospital District.

### Objective measurements of PA

The PA of the study participants was measured using three activity monitors (Hookie AM20, Polar Active, and Actigraph GT3X) continuously during a 2-week period. The activity measurement lasted for 15 days (including the day when the monitors were given). The monitors gave no feedback to the user.

Hookie AM20 (6.6 × 2.7 × 1.3 cm, mass 15 g, measurement range ± 16 g, Traxmeet Ltd., Espoo, Finland) is a triaxial accelerometer which can be worn on the hip on an elastic belt. Hookie AM20 collects raw acceleration data with a sampling frequency of 100 Hz. In this study, the epoch length was 30 s. For each epoch, MAD of the resultant acceleration was calculated from the raw acceleration data and converted to metabolic equivalents (METs) (Vähä-Ypyä et al., 2015a,b).

Polar Active (mass 45 g, frequency range 0.3–4.0 Hz, Polar Electro Ltd., Kempele, Finland) is a waterproof, wristwatch-like uniaxial accelerometer with a 21-day memory. The participants were advised to wear the device on the non-dominant hand for 24 h a day (except in the sauna bath). Using sex, age, body mass, and height as an input, Polar Active converts the measured acceleration signal to METs with the epoch length of 30 s. Polar Active has been shown to correlate well (*R*^2^ = 0.74) with the doubly labeled water technique while assessing EE during a 7-day period of military training (Kinnunen et al., 2012). A high correlation (*R*^2^ = 0.97) has also been obtained between EE measured with Polar Active prototype and indirect calorimetry during a 9.7-km hike (Brugniaux et al., 2010).

Actigraph GT3X (3.8 × 3.7 × 1.8 cm, mass 27 g, measurement range 0.05–2.5 g, Actigraph Inc., Pensacola, Florida, USA) is a hip-worn triaxial accelerometer which provides counts with the sampling frequency of 30 Hz. Actigraph has been widely used in large epidemiological studies (Robusto and Trost, 2012; Santos-Lozano et al., 2012; Troiano et al., 2014). During the data collection, the epoch length for Actigraph was 10 s which was transformed to 30 s for further analysis. Only the data from the vertical axis were included in the analysis. Previous studies have shown consistent results between vertical and triaxial Actigraph measurements (Kelly et al., 2013). Actigraph was worn on the same belt with the Hookie accelerometer. The participants were advised to wear the belt with both hip-worn activity monitors being on the right side during the waking hours, excluding the time spent in the sauna, bath, shower, or in other water activities.

The three PA measurement methods were compared within the time periods when all three devices had been simultaneously used. To be eligible for the analyses, all accelerometers had to be used simultaneously for at least four valid days; a valid day being at least 500 min of wearing time per day. The non-wearing time was defined as at least a 60-min period of consecutive zeros. Total between-subject daily time (in minutes) spent in sedentary time (ST), light (LPA), moderate (MPA), and vigorous (VPA) PA were compared.

For calculating the daily time spent on different PA levels, two different sets of MET-based thresholds were used for both MAD and Polar Active:

*(SET 1*) ST: ≤1.5 METs, LPA: 1.51–2.99 METs, MPA: 3–5.99 METs, and VPA: ≥6 METs; and
*(SET 2*) ST: <2 METs, LPA: 2–3.49 METs, MPA: 3.5–4.99 METs, and VPA: ≥5 METs.

The *SET 1* is widely used (Plasqui et al., 2013; Gibbs et al., 2015) whereas the *SET 2* is standard in Polar Active software and analyses. Actigraph counts were divided into MET-based PA classes using ≤100 counts/min for ST (Matthews et al., 2008) and the Freedson thresholds for LPA (101–1951 counts/min), MPA (1952–5724 counts/min), and VPA (≥5725 counts/min) (Freedson et al., 1998). The personal mean values over the period of valid data for each PA measurement method and activity level were calculated for the further analyses.

### Statistical analysis

The results were analyzed with IBM SPSS Statistics software (SPSS 19 for Windows, SPSS Inc., Chicago, Illinois). A *P*-value below 0.05 was considered statistically significant. At the method level, the mean time spent on different activity levels was calculated through personal mean values, and the between-method differences in ST, LPA, MPA, and VPA were analyzed using a repeated measures analysis of variance (ANOVA) test. If the assumption of sphericity was violated, the results with a Greenhouse-Geisser correction was presented. A post hoc test with Bonferroni adjustment for multiple comparisons was used. Cohen's *d*-values were used to determine the effect sizes.

Intra-class correlation coefficients (ICC) and Bland-Altman method were used for assessing agreement between the different methods at the individual level. In these analyses, the active time was defined as the sum of MPA and VPA, i.e., moderate-to-vigorous PA (MVPA). The correlation coefficient indicates the strength of association between two methods but the actual agreement is illustrated by the Bland-Altman method (Bland and Altman, 1986). The mean difference, standard deviation of the difference and 95% limits of agreement were calculated.

Receiver operating characteristics (ROC) graphs were used to evaluate and compare the used methods in classifying daily time spent in MVPA (Fawcett, 2006). Both MET thresholds for MVPA time, 3 and 3.5 METs, were included to the analysis. Each of the three methods was in turn used as a reference. The classification of a reference method was based on the fulfillment of PA recommendation which in this case was selected to be 60 min of MVPA time per day (Hallal et al., 2012). To estimate the discriminatory accuracy of each method, the area under the ROC curve (AUC) was calculated.

## Results

**Figure 1:**
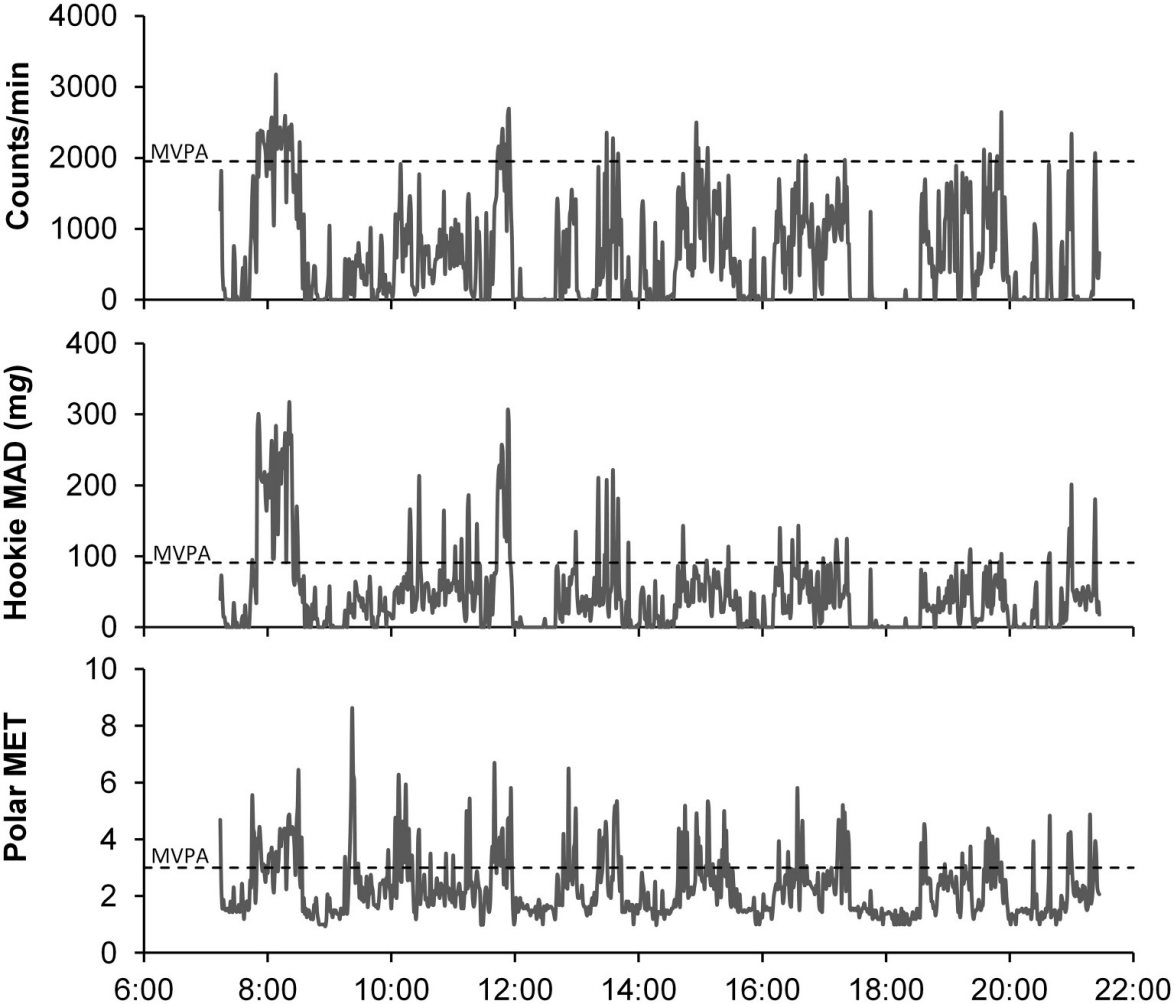
**An example of one-day activity measured with Polar Active, Hookie, and Actigraph**. MAD, mean amplitude deviation; MET, metabolic equivalent; MVPA, moderate-to-vigorous physical activity.

As a total, 376 valid days were included to the analyses. From these days, 98% exceeded also the limit of 600 min of wearing time per day. The mean number of valid days was 14 (SD 2.0) per person, and the mean wear time was 15.1 (SD 1.4) h/d. An example of 1-day activity data calculated to MAD (Hookie), METs (Polar Active), and Actigraph counts is presented in Figure 1. The daily patterns of activity shown as MAD and counts were rather similar. The MET values produced by Polar Active occasionally had the highest activity peaks at different time points compared to hip-worn devices.

**Figure 2:**
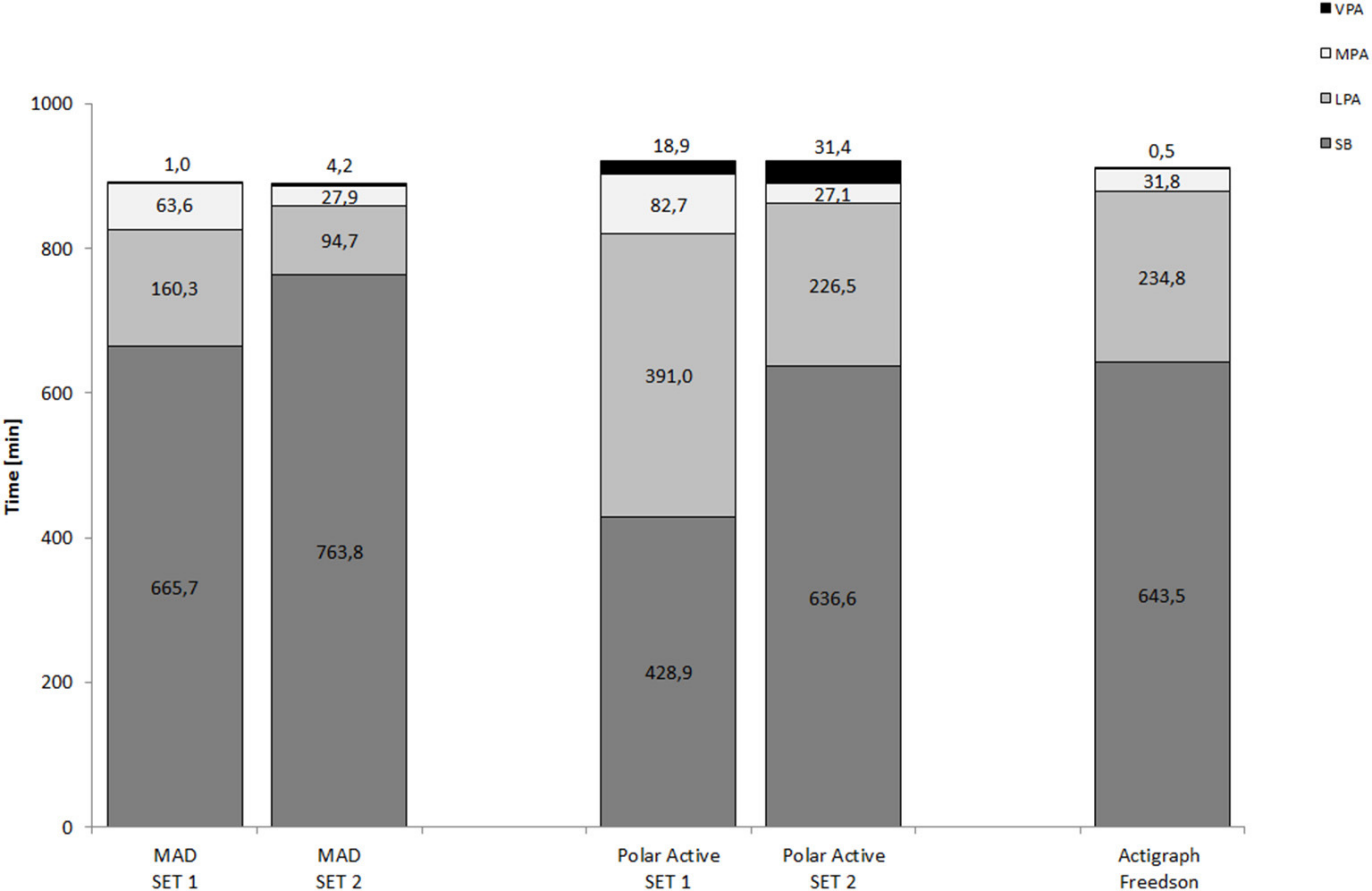
**Mean daily time (min/d) spent on different activity levels**. For MAD and Polar Active; (*SET 1*) ST: ≤1.5 METs, LPA: 1.51–2.99 METs, MPA: 3–5.99 METs, and VPA: ≥ 6 METs; and (*SET 2*) ST: <2 METs, LPA: 2–3.49 METs, MPA: 3.5–4.99 METs, and VPA: ≥5 METs. For Actigraph counts: ST ≤100 counts/min, LPA 101–1951 counts/min, MPA 1952–5724 counts/min, and VPA ≥ 5725 counts/min. The significant difference (*P* < 0.001, repeated measures ANOVA) between all measurement methods was found in all activity level except in MPA with the threshold 3.5–4.99 METs (*P* = 0.170). ST, sedentary time; LPA, light physical activity; MPA, moderate physical activity; VPA, vigorous physical activity.

The mean values for all four activity levels are shown in Figure 2, including the results based on the Freedson thresholds for Actigraph and both MET threshold sets (*SETs 1* and *2*) for MAD and Polar Active. When ST was analyzed with threshold <1.5 MET, values produced by MAD and Actigraph were closest to each other but yet differed significantly (mean difference 22.2 min/d, 95% CI 7.1–37.3, *P* < 0.01, Cohen's *d* = 0.238). Instead, with the threshold < 2 MET Polar Active and Actigraph gave similar results (mean difference 7.0 min/d, 95% CI 17.8–31.7, *P* = 1.0, *d* = 0.064).

Compared to the other two methods, the mean LPA time was the smallest analyzed by MAD with both thresholds (mean difference 74.5–230.7 min/d, *P* < 0.001, *d* = 1.399–3.726). The results of Polar Active and Actigraph were close to each other with the threshold 2–3.5 MET (mean difference 8.2 min/d, 95% CI 12.9–29.4, *P* = 0.985, *d* = 0.123). Instead, with the threshold 1.5–3 MET Polar Active resulted significantly higher amount of LPA compared to Actigraph (mean difference 156.2 min/d, 95% CI 131.9–180.5, *P* < 0.001, *d* = 2.315).

When the threshold 3–6 MET for MPA time was used, the value indicated by Polar Active was the highest, being 19.2 min/d higher than MAD (95% CI 5.8–32.6, *P* < 0.01, *d* = 0.733) and 51.0 min/d higher than Actigraph (95% CI 36.7–65.2, *P* < 0.001, *d* = 2.175). Using the threshold 3.5–5 MET, the results were close to each other with all three accelerometers [*F*_(1.44, 37.43)_ = 1.92, *P* = 0.170].

The mean VPA time was the highest measured with Polar Active compared to MAD and Actigraph with both threshold sets (mean difference 17.9–30.9 min/d, *P* < 0.001, *d* = 1.313–2.020). For Polar Active the difference in results between the threshold *SET 1* and *2* was 13 min/d (*d* = 1.313). The results for VPA were close to zero produced by MAD with the threshold >6 MET and by Actigraph with the Freedson threshold (1.0 vs. 0.5 min/d, *P* = 0.526, *d* = 0.335). With the threshold >5 MET MAD resulted in 4 min difference to Actigraph (95% CI 0.8–6.5, *P* < 0.01, *d* = 0.884).

**Figure 3:**
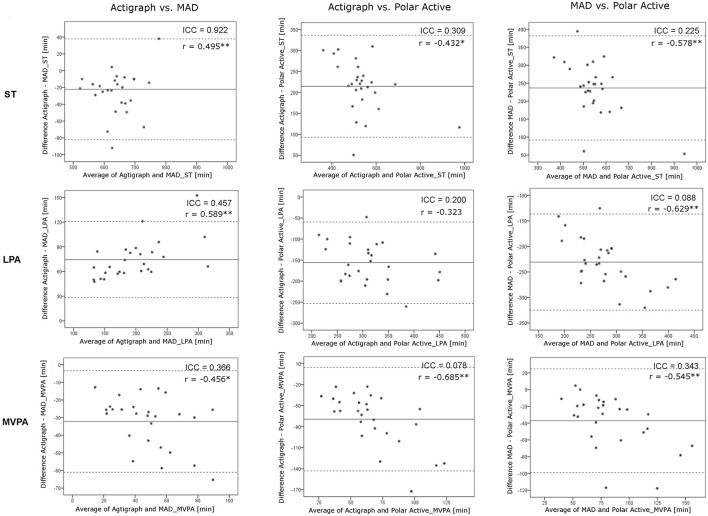
**The agreement between methods at time spent on different activity levels**. ST: ≤1.5 METs, LPA: 1.51–2.99 METs, MVPA: ≥3 METs, ICC, intra-class correlation coefficient; MET, metabolic equivalent; ST, sedentary time; LPA, light physical activity; MVPA, moderate-to-vigorous physical activity. ^*^p < 0.05, ^**^p < 0.01 for the significance level of correlation coefficients.

The Bland-Altman plots illustrating the agreement between different methods in estimating time spent on different activity levels (analyzed with the threshold *SET 1*) are shown in Figure 3. The highest agreement was found between MAD and Actigraph on ST (ICC = 0.922). For MVPA with the threshold > 3 MET, all measurement methods gave divergent results. Polar Active overestimated time spent in MVPA by 37.1 min/d (SD 31.6 min/d) compared to MAD and by 69.3 min/d (SD 37.7 min/d) compared to Actigraph.

**Figure 4:**
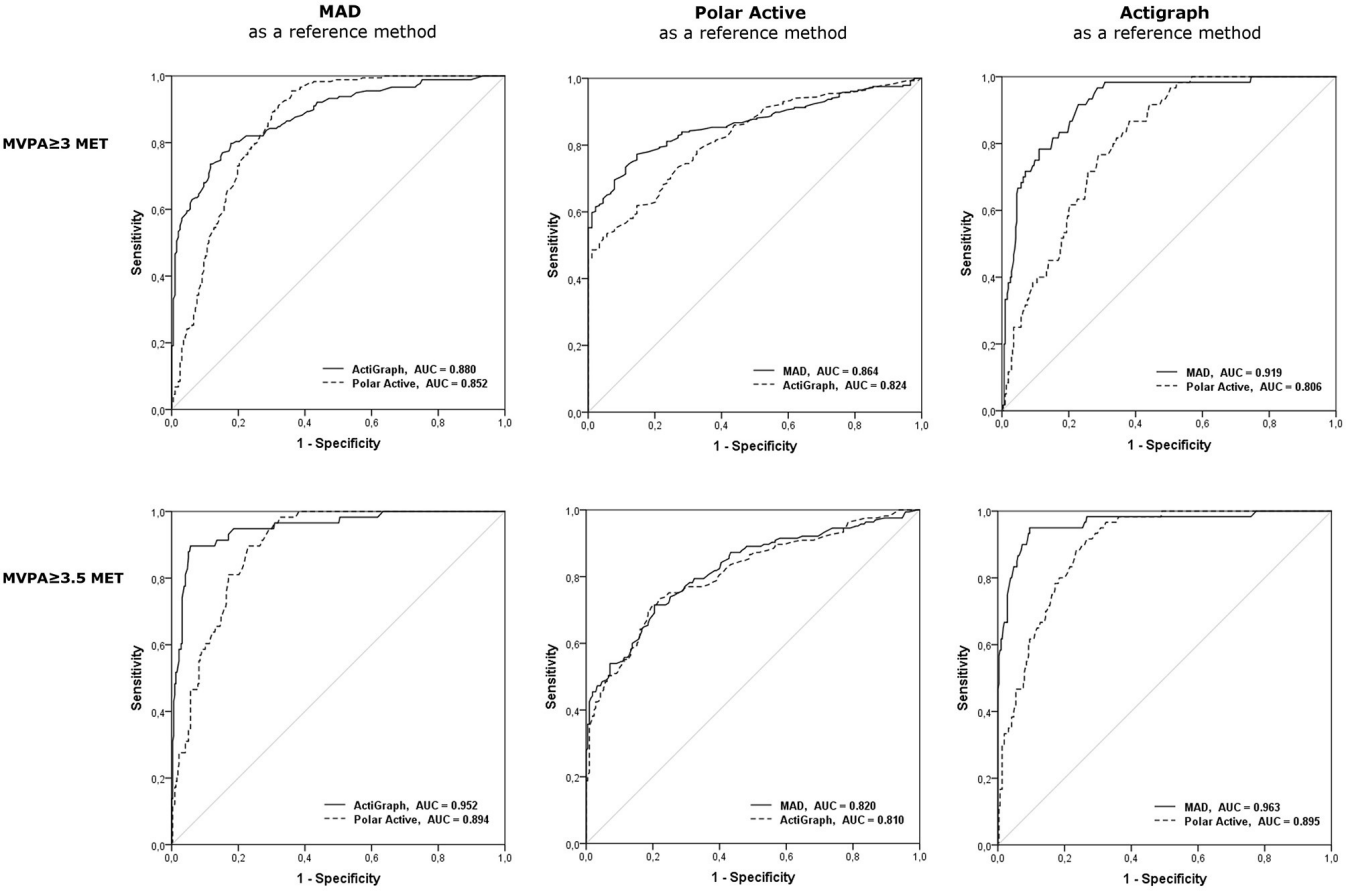
**The comparisons of the fulfillment of MVPA recommendation (60 min daily) measured by MAD, Polar Active, and Actigraph with the Freedson threshold**. Two MET thresholds for MVPA time, 3 and 3.5 METs, are included. AUC, area under the curve; MET, metabolic equivalent; MVPA, moderate-to-vigorous physical activity.

The ROC curves for comparisons of the fulfillment of MVPA recommendation measured by MAD, Polar Active, and Actigraph with the Freedson thresholds are shown in Figure 4. Moderate to high agreement between all the methods was found, the best degree of consistency observed with the threshold value of 3.5 MET, Actigraph being the reference method [AUC for MAD = 0.963 (95% CI 0.934–0.991), AUC for Polar Active = 0.895 (95% CI 0.860–0.929)].

## Discussion

In this study, the agreement in time spent on different PA intensity levels calculated using MAD for triaxial raw acceleration data, Polar Active, and the Freedson equation for Actigraph counts was examined among middle-aged people. The findings showed that in daily life conditions the agreement between the used methods varied by the selected activity thresholds as well as by the intensity of PA. However, according to the ROC analyses, there is moderate to high agreement for the fulfillment of the recommendation for daily MVPA of 60 min.

Polar Active resulted in the lowest amount of ST and the highest amount of MVPA with both MET threshold sets for differentiating between different PA levels. The results of Polar Active were closer to the results of the two other methods when the standard Polar Active thresholds were used instead of the widely used threshold *SET* 1 (Figures 2, 4). The level of overestimation increased in wrist-worn Polar Active compared to the two hip-worn methods with higher values of accumulated MVPA time (significant magnitude-associations in Bland-Altman plots in Figure 3). The difference between the methods in MVPA time was most evident for the vigorous activity. The difference was also revealed in the daily pattern of activity (Figure 1). These results are in accordance with the previous findings stating that wrist-worn monitors show higher activity output than monitors worn on hip (Hildebrand et al., 2014). When indirect calorimetry has been used as the criterion measure, hip-worn monitors have been shown to be more accurate in comparison to wrist-worn monitors (Esliger et al., 2011; Rosenberger et al., 2013). However, recent studies showed that the outcomes are very similar between hip and wrist when appropriate site-specific equations are used (Trost et al., 2014; Crouter et al., 2015).

Deviating results in some examined activity levels were observed also between MAD and Actigraph based on the Freedson thresholds. MAD increasingly underestimated LPA but overestimated MVPA compared to Actigraph (significant magnitude-associations in Bland-Altman plots in Figure 3). However, comparable results between the devices might have been achieved if the raw Actigraph data had been analyzed with the universal MAD approach (Aittasalo et al., 2015; Vähä-Ypyä et al., 2015b). However, due to the long-term data collection, raw data were not available for the Actigraph device.

The thresholds used may exert even more effect on results than the monitor location (Rowlands et al., 2014). The Freedson thresholds have been stated to overestimate resting and LPA by 13% and underestimate MPA by 60%, compared to the results from the portable metabolic measurement system (Strath et al., 2003). These thresholds were developed based on treadmill walking and running which might be one reason for the difference in free-living conditions (Freedson et al., 1998). In the present study the thresholds developed by Troiano et al., 2008 were additionally tested, resulting in similar values compared to the results analyzed with the Freedson thresholds (data not shown; Troiano et al., 2008).

The ROC analyses showed moderate to high agreement between the different methods for the fulfillment of the recommendation for daily MVPA of 60 min, which suggests that any of the methods can be used to identify persons with insufficient daily amount of MVPA. However, the used method and threshold affected the level of agreement (Figure 4).

In this study, we did not use indirect calorimetry or doubly labeled water technique as a reference method, which may be considered a limitation. However, these techniques cannot reveal specific patterns of PA or sedentary behavior but rather provide the mean EE over the period of interest. It is important to note that even if in some cases the consistency between devices was evident at a group level, differences at an individual level could still be quite large. Additionally, the sample presented a narrow age range of middle-aged men and women, limiting the generalizability of the findings particularly to children or older adults.

The main strength of this study was the long 2-week measurement period in free-living environment. The monitors were conscientiously used by the study participants, the mean wear time satisfactorily covering the waking hours as well as the 2-week measurement period.

## Conclusion

This study revealed that in daily life conditions the agreement between the MAD of raw triaxial acceleration, Polar Active, and Actigraph with the Freedson thresholds for measuring time spent on different activity levels in middle-aged people is highly dependent on the thresholds used as well as on the intensity level of physical activity. Good agreement between the methods was found for the fulfillment of the daily recommendation for MVPA of 60 min. However, based on the current results, it is not possible to say which of the methods is superior to each other. Instead, this study showed that without additional statistical adjustment, the results obtained from these types of accelerometers and analyses cannot be used interchangeably when estimating time spent on different activity levels on daily life. Anyway, any of the methods can be used to identify persons with insufficient daily amount of MVPA.

## Author contributions

RA, TJ, TT, RK, SKK, JA, KHH and AML have given significant contribution for the planning and implementation of the study, and for the planning and writing of the submitted manuscript. TJ, RK and SKK have been the principal investigators and have obtained funding for the study. JK and HH have been responsible for analyzing data from Polar Active and Actigraph. HVY and HS have been responsible for performing MAD analysis. The corresponding author AML has been responsible for statistical analysis and writing the first draft of the manuscript. All authors have revised, read, and accepted the final version of the manuscript.

### Conflict of interest statement

RA is currently (from November 2015) employed by Polar Electro Ltd. She was not involved in the company during data collection and analysis. The company had no role in the conduct of the study or decision to publish. The other authors declare that the research was conducted in the absence of any commercial or financial relationships that could be construed as a potential conflict of interest. The reviewer WZ and handling Editor declared their shared affiliation, and the handling Editor states that the process nevertheless met the standards of a fair and objective review.
